# *catena*-Poly[[bis­(pyridine-κ*N*)zinc(II)]-μ-benzene-1,4-dicarboxyl­ato-κ^2^ *O* ^1^:*O* ^4^]

**DOI:** 10.1107/S1600536810022385

**Published:** 2010-06-23

**Authors:** Li-Fen Wang, Chuan-Qiang Li, Wen-Ge Qiu, Hong He

**Affiliations:** a College of Environmental and Energy Engineering, Beijing University of Technology, Beijing 100124, People’s Republic of China

## Abstract

In the title coordination polymer, [Zn(C_8_H_4_O_4_)(C_5_H_5_N)_2_]_*n*_, the Zn^II^ atom, located on a twofold rotation axis, is tetra­coordinated by two monodentate O atoms from two different carboxyl­ate groups and two pyridyl N atoms, forming a distorted tetra­hedral geometry. The Zn^II^ atoms are bridged by terephthalate ligands, generating an infinite zigzag chain along [101].

## Related literature

For related structures, see: Li *et al.* (2007 ▶); Mori *et al.* (2004 ▶).
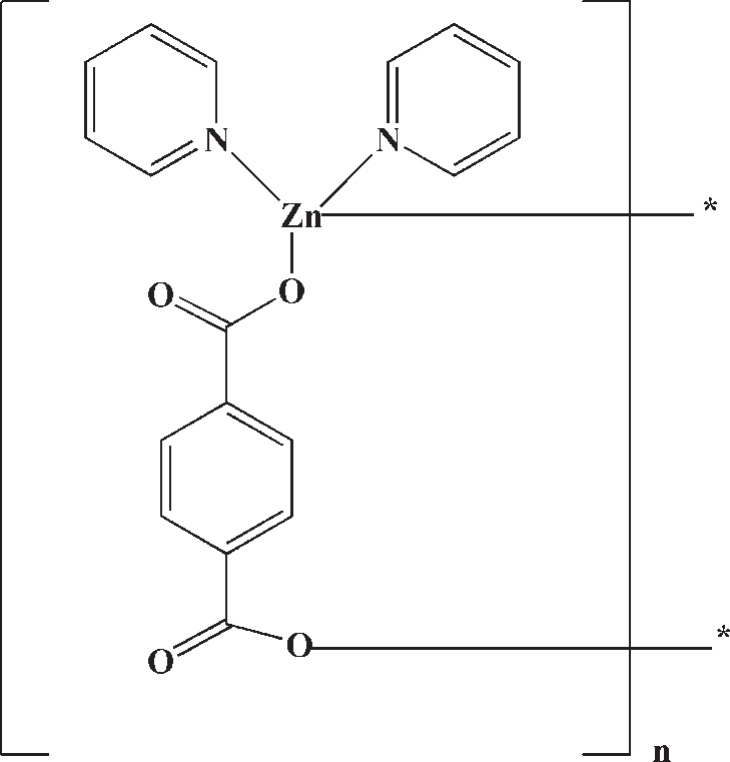


## Experimental

### 

#### Crystal data

[Zn(C_8_H_4_O_4_)(C_5_H_5_N)_2_]
*M* *_r_* = 387.68
Monoclinic, 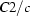

*a* = 20.054 (8) Å
*b* = 6.299 (2) Å
*c* = 14.761 (6) Å
β = 111.500 (6)°
*V* = 1734.9 (11) Å^3^
*Z* = 4
Mo *K*α radiation
μ = 1.44 mm^−1^
*T* = 173 K
0.24 × 0.20 × 0.15 mm

#### Data collection

Bruker SMART CCD area-detector diffractometer
Absorption correction: numerical (*SADABS*; Bruker, 1998 ▶) *T*_min_ = 0.724, *T*_max_ = 0.813
7306 measured reflections
1975 independent reflections
1915 reflections with *I* > 2σ(*I*)
*R*_int_ = 0.039

#### Refinement

*R*[*F* ^2^ > 2σ(*F* ^2^)] = 0.036
*wR*(*F* ^2^) = 0.082
*S* = 1.03
1975 reflections
114 parameters
H-atom parameters constrained
Δρ_max_ = 0.35 e Å^−3^
Δρ_min_ = −0.22 e Å^−3^

### 

Data collection: *SMART* (Bruker, 1998 ▶); cell refinement: *SAINT* (Bruker, 1998 ▶); data reduction: *SAINT*; program(s) used to solve structure: *SHELXS97* (Sheldrick, 2008 ▶); program(s) used to refine structure: *SHELXL97* (Sheldrick, 2008 ▶); molecular graphics: *SHELXTL* (Sheldrick, 2008 ▶); software used to prepare material for publication: *SHELXTL* (Sheldrick, 2008 ▶).

## Supplementary Material

Crystal structure: contains datablocks global, I. DOI: 10.1107/S1600536810022385/is2548sup1.cif

Structure factors: contains datablocks I. DOI: 10.1107/S1600536810022385/is2548Isup2.hkl

Additional supplementary materials:  crystallographic information; 3D view; checkCIF report

## Figures and Tables

**Fig. 1.**
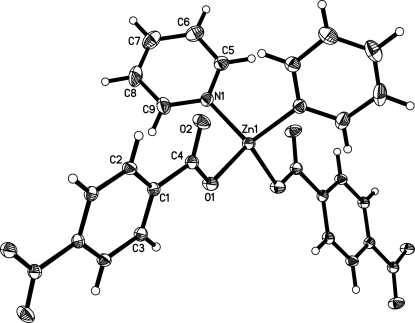
A view of the molecular structure of the title compound, with displacement ellipsoids drawn at the 30% probability level. Hydrogen atoms have been omitted for clarity.

**Fig. 2.**
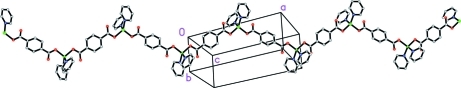
An illustration of the zigzag chain formed by bridging terephthalate ligands. H atoms have been omitted.

## supplementary crystallographic information

### Comment

A great number of the crystal structures of one-dimensional chain complexes have been extensively investigated (Li *et al.* 2007; Mori *et al.* 2004), in most of which interchain hydrogen bonds or π–π interactions connect the chains to produce two-dimensional or three-dimensional structures. Here, we report the synthesis and crystal structure of a new one-dimensional zigzag coordination polymer.

In the title coordination polymer, each Zn(II) atom is four-coordinated. The coordination environment around the Zn(II) ions represents a slightly distorted tetrahedral geometry with two pyridyl N and two monodentate O atoms from two different carboxylates. The Zn centers are interconnected by terephthalate ligands to form an infinite zigzag chain. The Zn—O bond distance between Zn(II) and carboxylate O atom is 1.9622 (18) Å, and the Zn—N bond distance between Zn(II) and the N atom of the pyridine is 2.038 (2) Å.

### Experimental

A solution containing a 2:1 molar ratio of 1,4-benzenedicarboxylic acid (0.022 g) and zinc nitrate hexahydrate (0.041 g) in a mixture of pyridine (2 ml) and *N*,*N*-dimethylformamide (2 ml) was sealed in a 5 ml transparent vitreous reactor and kept at 343 K for 5 days, and then cooled to room temperature. The mixture was filtered and colorless crystals suitable for the X-ray investigation were collected.

### Refinement

All H atoms were positioned geometrically (C—H = 0.95 Å) and treated as riding, with *U*_iso_(H) = 1.2*U*_eq_(C).

### Crystal data

**Table:**

[Zn(C_8_H_4_O_4_)(C_5_H_5_N)_2_]	*F*(000) = 792
*M**_r_* = 387.68	*D*_x_ = 1.484 Mg m^−^^3^
Monoclinic, *C*2/*c*	Mo *K*α radiation, λ = 0.71073 Å
Hall symbol: -C 2yc	Cell parameters from 968 reflections
*a* = 20.054 (8) Å	θ = 2.2–27.5°
*b* = 6.299 (2) Å	µ = 1.44 mm^−^^1^
*c* = 14.761 (6) Å	*T* = 173 K
β = 111.500 (6)°	Block, colorless
*V* = 1734.9 (11) Å^3^	0.24 × 0.20 × 0.15 mm
*Z* = 4	

### Data collection

**Table:**

Bruker SMART CCD area-detector diffractometer	1975 independent reflections
Radiation source: sealed tube	1915 reflections with *I* > 2σ(*I*)
graphite	*R*_int_ = 0.039
ω scans	θ_max_ = 27.5°, θ_min_ = 3.0°
Absorption correction: numerical (*SADABS*; Bruker, 1998)	*h* = −26→19
*T*_min_ = 0.724, *T*_max_ = 0.813	*k* = −8→8
7306 measured reflections	*l* = −19→19

### Refinement

**Table:**

Refinement on *F*^2^	Primary atom site location: structure-invariant direct methods
Least-squares matrix: full	Secondary atom site location: difference Fourier map
*R*[*F*^2^ > 2σ(*F*^2^)] = 0.036	Hydrogen site location: inferred from neighbouring sites
*wR*(*F*^2^) = 0.082	H-atom parameters constrained
*S* = 1.03	*w* = 1/[σ^2^(*F*_o_^2^) + (0.030*P*)^2^ + 3.5*P*] where *P* = (*F*_o_^2^ + 2*F*_c_^2^)/3
1975 reflections	(Δ/σ)_max_ < 0.001
114 parameters	Δρ_max_ = 0.35 e Å^−^^3^
0 restraints	Δρ_min_ = −0.22 e Å^−^^3^

### Special details

**Table:**

Geometry. All e.s.d.'s (except the e.s.d. in the dihedral angle between two l.s. planes) are estimated using the full covariance matrix. The cell e.s.d.'s are taken into account individually in the estimation of e.s.d.'s in distances, angles and torsion angles; correlations between e.s.d.'s in cell parameters are only used when they are defined by crystal symmetry. An approximate (isotropic) treatment of cell e.s.d.'s is used for estimating e.s.d.'s involving l.s. planes.
Refinement. Refinement of *F*^2^ against ALL reflections. The weighted *R*-factor *wR* and goodness of fit *S* are based on *F*^2^, conventional *R*-factors *R* are based on *F*, with *F* set to zero for negative *F*^2^. The threshold expression of *F*^2^ > 2 σ (*F*^2^) is used only for calculating *R*-factors (gt) *etc*. and is not relevant to the choice of reflections for refinement. *R*-factors based on *F*^2^ are statistically about twice as large as those based on *F*, and *R*- factors based on ALL data will be even larger.

### Fractional atomic coordinates and isotropic or equivalent isotropic displacement parameters (Å^2^)

**Table:**

	*x*	*y*	*z*	*U*_iso_*/*U*_eq_	
Zn1	0.0000	0.39350 (5)	0.2500	0.02669 (12)	
O1	0.07418 (8)	0.5959 (2)	0.32459 (12)	0.0340 (4)	
O2	0.12091 (8)	0.3122 (3)	0.41504 (13)	0.0409 (4)	
N1	0.04941 (9)	0.1981 (3)	0.18419 (13)	0.0300 (4)	
C1	0.18900 (10)	0.6311 (3)	0.44879 (15)	0.0245 (4)	
C2	0.24474 (11)	0.5421 (3)	0.52709 (15)	0.0271 (4)	
H2	0.2410	0.3996	0.5457	0.032*	
C3	0.19468 (11)	0.8406 (3)	0.42210 (15)	0.0265 (4)	
H3	0.1570	0.9028	0.3691	0.032*	
C4	0.12371 (11)	0.5003 (3)	0.39400 (15)	0.0271 (4)	
C5	0.02091 (13)	0.0111 (4)	0.14623 (17)	0.0343 (5)	
H5	−0.0249	−0.0258	0.1465	0.041*	
C6	0.05508 (15)	−0.1297 (4)	0.10692 (19)	0.0456 (6)	
H6	0.0332	−0.2612	0.0809	0.055*	
C7	0.12141 (17)	−0.0777 (5)	0.1057 (2)	0.0539 (8)	
H7	0.1460	−0.1720	0.0784	0.065*	
C8	0.15136 (16)	0.1135 (5)	0.1447 (2)	0.0543 (8)	
H8	0.1971	0.1532	0.1447	0.065*	
C9	0.11451 (13)	0.2473 (4)	0.18390 (19)	0.0411 (6)	
H9	0.1359	0.3783	0.2115	0.049*	

### Atomic displacement parameters (Å^2^)

**Table:**

	*U*^11^	*U*^22^	*U*^33^	*U*^12^	*U*^13^	*U*^23^
Zn1	0.01866 (18)	0.02270 (19)	0.0321 (2)	0.000	0.00149 (14)	0.000
O1	0.0219 (7)	0.0257 (8)	0.0404 (9)	−0.0005 (6)	−0.0049 (6)	0.0005 (7)
O2	0.0300 (8)	0.0289 (9)	0.0483 (10)	−0.0076 (7)	−0.0041 (7)	0.0063 (7)
N1	0.0251 (9)	0.0318 (10)	0.0317 (10)	0.0023 (7)	0.0087 (7)	0.0032 (8)
C1	0.0182 (9)	0.0249 (10)	0.0265 (10)	−0.0011 (7)	0.0036 (8)	−0.0023 (8)
C2	0.0231 (10)	0.0205 (9)	0.0318 (11)	0.0005 (8)	0.0033 (8)	0.0014 (8)
C3	0.0190 (9)	0.0270 (10)	0.0274 (10)	0.0024 (8)	0.0011 (8)	0.0021 (8)
C4	0.0200 (10)	0.0258 (11)	0.0299 (11)	−0.0003 (8)	0.0025 (8)	−0.0010 (9)
C5	0.0328 (12)	0.0342 (12)	0.0334 (12)	0.0020 (9)	0.0090 (10)	−0.0010 (10)
C6	0.0526 (16)	0.0428 (15)	0.0383 (13)	0.0090 (12)	0.0131 (12)	−0.0037 (11)
C7	0.0598 (19)	0.063 (2)	0.0457 (16)	0.0222 (15)	0.0280 (14)	0.0043 (14)
C8	0.0421 (15)	0.075 (2)	0.0576 (18)	0.0072 (14)	0.0328 (14)	0.0088 (16)
C9	0.0349 (13)	0.0465 (15)	0.0450 (14)	−0.0031 (11)	0.0185 (11)	0.0069 (12)

### Geometric parameters (Å, °)

**Table:**

Zn1—O1	1.9621 (15)	C3—C2^i^	1.384 (3)
Zn1—N1	2.0363 (19)	C3—H3	0.9500
O1—C4	1.286 (2)	C5—C6	1.372 (3)
O2—C4	1.231 (3)	C5—H5	0.9500
N1—C5	1.339 (3)	C6—C7	1.377 (4)
N1—C9	1.343 (3)	C6—H6	0.9500
C1—C3	1.394 (3)	C7—C8	1.374 (4)
C1—C2	1.397 (3)	C7—H7	0.9500
C1—C4	1.507 (3)	C8—C9	1.380 (4)
C2—C3^i^	1.384 (3)	C8—H8	0.9500
C2—H2	0.9500	C9—H9	0.9500
			
O1—Zn1—O1^ii^	98.96 (9)	O2—C4—O1	123.93 (19)
O1—Zn1—N1^ii^	121.77 (8)	O2—C4—C1	120.10 (18)
O1—Zn1—N1	105.02 (8)	O1—C4—C1	115.94 (18)
O1^ii^—Zn1—N1	121.77 (8)	N1—C5—C6	122.9 (2)
N1^ii^—Zn1—N1	105.60 (11)	N1—C5—H5	118.6
C4—O1—Zn1	110.39 (13)	C6—C5—H5	118.6
C5—N1—C9	117.9 (2)	C5—C6—C7	119.1 (3)
C5—N1—Zn1	121.59 (15)	C5—C6—H6	120.4
C9—N1—Zn1	120.36 (17)	C7—C6—H6	120.4
C3—C1—C2	119.33 (19)	C8—C7—C6	118.6 (3)
C3—C1—C4	120.72 (18)	C8—C7—H7	120.7
C2—C1—C4	119.95 (19)	C6—C7—H7	120.7
C3^i^—C2—C1	120.8 (2)	C7—C8—C9	119.5 (3)
C3^i^—C2—H2	119.6	C7—C8—H8	120.3
C1—C2—H2	119.6	C9—C8—H8	120.3
C2^i^—C3—C1	119.91 (19)	N1—C9—C8	122.1 (3)
C2^i^—C3—H3	120.0	N1—C9—H9	119.0
C1—C3—H3	120.0	C8—C9—H9	119.0

Symmetry codes: (i) −*x*+1/2, −*y*+3/2, −*z*+1; (ii) −*x*, *y*, −*z*+1/2.
